# 2,3-Diphenyl-1,3-thia­zolidin-4-one

**DOI:** 10.1107/S1600536814015128

**Published:** 2014-07-02

**Authors:** Hemant P. Yennawar, John Tierney, Lee J. Silverberg

**Affiliations:** a Department of Chemistry, Pennsylvania State University, University Park, PA 16802, USA; b Pennsylvania State University, Brandywine Campus, 312 M Main Building, 25 Yearsley Mill Rd, Media, PA 19063, USA; c Pennsylvania State University, Schuylkill Campus, 200 University Drive, Schuylkill Haven, PA 17972, USA

**Keywords:** crystal structure

## Abstract

The title compound, C_15_H_13_NOS, is a chiral mol­ecule crystallized as a racemate, with two molecules in the asymmetric unit. In each of the mol­ecules, the five-membered thia­zine ring has an envelope conformation, with the S atom forming the flap. In one mol­ecule, the angle between the two phenyl-ring planes is 82.77 (7)°, while in the other it is 89.12 (6)°. In the crystal, mol­ecules are linked into chains along the *b-*axis direction by C—H⋯O hydrogen bonds.

## Related literature   

For the preparation of the title compound, see: Tierney (1989 ▶). For the crystal structure of a tin complex of the title compound, see: Smith *et al.* (1995 ▶). For the synthesis and crystal structures of related compounds, see: Yennawar & Silverberg (2013 ▶, 2014 ▶); Fun *et al.* (2011 ▶). For reviews on 1,3-thia­zolidin-4-ones, see: Brown (1961 ▶); Singh *et al.* (1981 ▶); Metally *et al.* (2006 ▶); Abhishek *et al.* (2012 ▶).
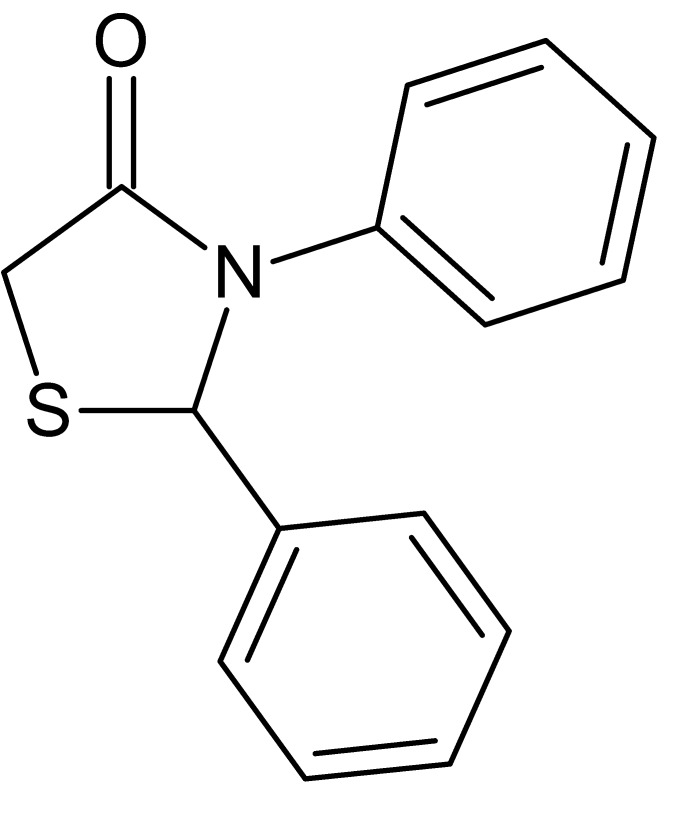


## Experimental   

### 

#### Crystal data  

C_15_H_13_NOS
*M* *_r_* = 255.32
Monoclinic, 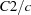

*a* = 32.413 (13) Å
*b* = 6.196 (3) Å
*c* = 25.964 (11) Å
β = 100.258 (7)°
*V* = 5131 (4) Å^3^
*Z* = 16
Mo *K*α radiation
μ = 0.24 mm^−1^
*T* = 298 K
0.14 × 0.12 × 0.08 mm

#### Data collection  

Bruker SMART APEX CCD diffractometer
Absorption correction: multi-scan (*SADABS*; Bruker, 2001 ▶) *T*_min_ = 0.807, *T*_max_ = 0.981
23146 measured reflections
6334 independent reflections
5015 reflections with *I* > 2σ(*I*)
*R*_int_ = 0.028

#### Refinement  

*R*[*F* ^2^ > 2σ(*F* ^2^)] = 0.049
*wR*(*F* ^2^) = 0.147
*S* = 1.01
6334 reflections
325 parameters
H-atom parameters not refined
Δρ_max_ = 0.33 e Å^−3^
Δρ_min_ = −0.22 e Å^−3^

### 

Data collection: *SMART* (Bruker, 2001 ▶); cell refinement: *SAINT* (Bruker, 2001 ▶); data reduction: *SAINT*; program(s) used to solve structure: *SHELXS97* (Sheldrick, 2008 ▶); program(s) used to refine structure: *SHELXL97* (Sheldrick, 2008 ▶); molecular graphics: *XSHELL* (Bruker, 2001 ▶) and *ORTEP-3 for Windows* (Farrugia, 2012 ▶); software used to prepare material for publication: *SHELXL97*.

## Supplementary Material

Crystal structure: contains datablock(s) I. DOI: 10.1107/S1600536814015128/fy2114sup1.cif

Structure factors: contains datablock(s) I. DOI: 10.1107/S1600536814015128/fy2114Isup2.hkl

Supporting information file. DOI: 10.1107/S1600536814015128/fy2114Isup3.mol

Supporting information file. DOI: 10.1107/S1600536814015128/fy2114Isup4.cml

CCDC reference: 1010627

Additional supporting information:  crystallographic information; 3D view; checkCIF report

## Figures and Tables

**Table 1:** Hydrogen-bond geometry (Å, °)

*D*—H⋯*A*	*D*—H	H⋯*A*	*D*⋯*A*	*D*—H⋯*A*
C15—H15⋯O1^i^	0.93	2.58	3.470 (2)	160
C1—H1⋯O1^i^	0.98	2.49	3.466 (2)	172
C16—H16⋯O2^ii^	0.98	2.34	3.301 (3)	168
C17—H17*B*⋯O2^iii^	0.97	2.41	3.313 (3)	155

Symmetry codes: (i) 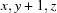; (ii) 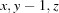; (iii) 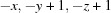.

**Fig. 1.**
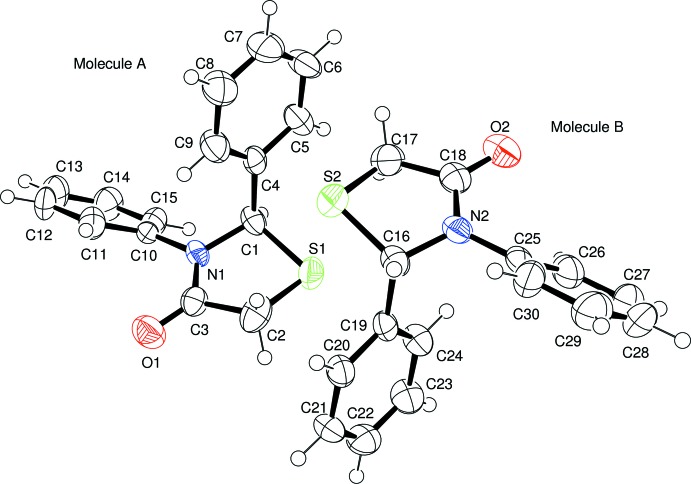
ORTEP view of the title comound. Thermal ellipsoids are drawn at 50% probability.

**Fig. 2.**
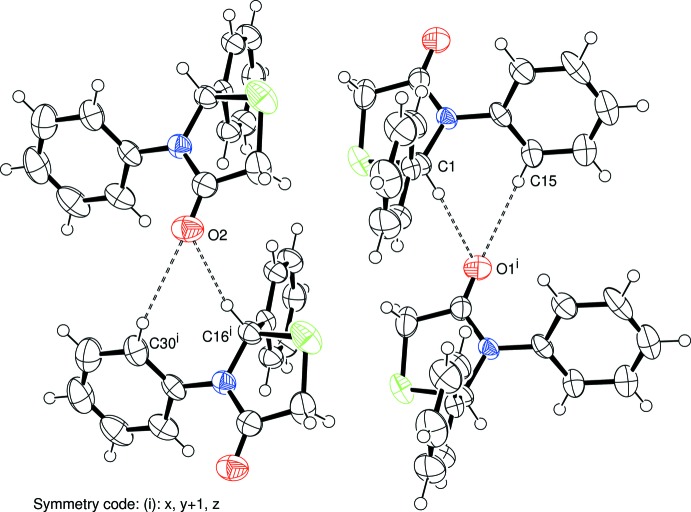
View of b-c plane with C—H···O interactions shown as dashed lines.

**Fig. 3.**
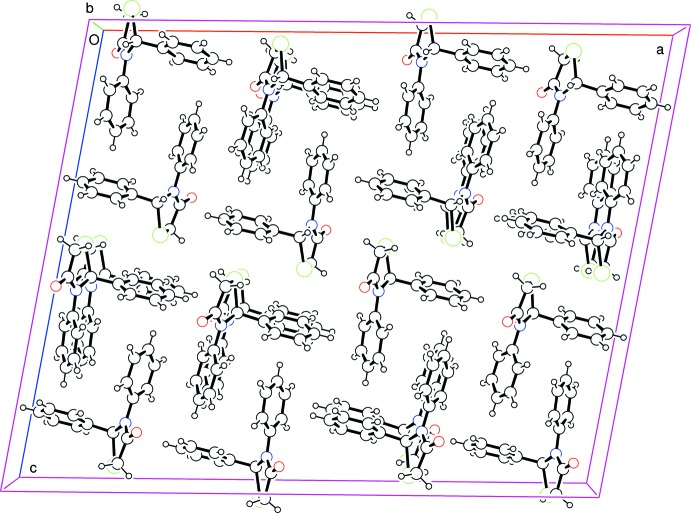
Unit-cell contents.

## supplementary crystallographic information

### S1. Comment

We have recently reported the syntheses and crystal structures of 6,7-diphenyl-5-thia-7-azaspiro[2.6]nonan-8-one, a seven-membered heterocycle (Yennawar and Silverberg, 2013), and 2,3-diphenyl-2,3,5,6-tetrahydro-4*H*-1,3-thiazin-4-one, a similar six-membered heterocycle (Yennawar and Silverberg, 2014). We report here the crystal structure of 2,3-diphenyl-1,3-thiazolidin-4-one (Tierney, 1989), the analogous five-membered heterocycle. The crystal structure of a tin complex of the title compound has been previously reported (Smith *et al.*, 1995), but the structure of the title compound has not. The crystal structure of similar compound 3-benzyl-2-phenyl-1,3-thiazolidin-4-one has been reported (Fun *et al.*, 2011). The 1,3-thiazolidin-4-ones are an important class of compounds with a wide range of biological activity (Brown, 1961; Singh, *et al.*, 1981; Metally *et al.*, 2006; Abhishek *et al.*, 2012).

### S2. Experimental

A sample of the title compound, prepared according to Tierney (1989), was recrystallized from ethanol. *R**_f_* = 0.54 (50% EtOAc/hexanes). m.p.: 131–133°C (lit. 131–132°C). Crystals for X-ray crystallography were grown by slow evaporation from toluene.

### S3. Refinement

The C-bound H atoms were geometrically placed with C—H = 0.93–0.97 Å, and refined as riding with *U*_iso_(H) = 1.2U_eq_(C).

### Crystal data

**Table:**

C_15_H_13_NOS	*F*(000) = 2144
*M**_r_* = 255.32	*D*_x_ = 1.322 Mg m^−^^3^
Monoclinic, *C*2/*c*	Melting point: 405(1) K
Hall symbol: -C 2yc	Mo *K*α radiation, λ = 0.71073 Å
*a* = 32.413 (13) Å	Cell parameters from 7831 reflections
*b* = 6.196 (3) Å	θ = 2.2–28.3°
*c* = 25.964 (11) Å	µ = 0.24 mm^−^^1^
β = 100.258 (7)°	*T* = 298 K
*V* = 5131 (4) Å^3^	Block, colorless
*Z* = 16	0.14 × 0.12 × 0.08 mm

### Data collection

**Table:**

Bruker SMART APEX CCD diffractometer	6334 independent reflections
Radiation source: fine-focus sealed tube	5015 reflections with *I* > 2σ(*I*)
Graphite monochromator	*R*_int_ = 0.028
Detector resolution: 8.34 pixels mm^-1^	θ_max_ = 28.3°, θ_min_ = 1.6°
phi and ω scans	*h* = −42→42
Absorption correction: multi-scan (*SADABS*; Bruker, 2001)	*k* = −8→8
*T*_min_ = 0.807, *T*_max_ = 0.981	*l* = −34→30
23146 measured reflections	

### Refinement

**Table:**

Refinement on *F*^2^	Primary atom site location: structure-invariant direct methods
Least-squares matrix: full	Secondary atom site location: difference Fourier map
*R*[*F*^2^ > 2σ(*F*^2^)] = 0.049	Hydrogen site location: inferred from neighbouring sites
*wR*(*F*^2^) = 0.147	H-atom parameters not refined
*S* = 1.01	*w* = 1/[σ^2^(*F*_o_^2^) + (0.0897*P*)^2^ + 1.5154*P*] where *P* = (*F*_o_^2^ + 2*F*_c_^2^)/3
6334 reflections	(Δ/σ)_max_ < 0.001
325 parameters	Δρ_max_ = 0.33 e Å^−^^3^
0 restraints	Δρ_min_ = −0.22 e Å^−^^3^

### Special details

**Table:**

Experimental. The data collection nominally covered a full sphere of reciprocal space by a combination of 4 sets of ω scans each set at different φ and/or 2θ angles and each scan (30 s exposure) covering -0.300° degrees in ω. The crystal to detector distance was 5.82 cm.
Geometry. All e.s.d.'s (except the e.s.d. in the dihedral angle between two l.s. planes) are estimated using the full covariance matrix. The cell e.s.d.'s are taken into account individually in the estimation of e.s.d.'s in distances, angles and torsion angles; correlations between e.s.d.'s in cell parameters are only used when they are defined by crystal symmetry. An approximate (isotropic) treatment of cell e.s.d.'s is used for estimating e.s.d.'s involving l.s. planes.
Refinement. Refinement of *F*^2^ against ALL reflections. The weighted *R*-factor *wR* and goodness of fit *S* are based on *F*^2^, conventional *R*-factors *R* are based on *F*, with *F* set to zero for negative *F*^2^. The threshold expression of *F*^2^ > σ(*F*^2^) is used only for calculating *R*-factors(gt) *etc*. and is not relevant to the choice of reflections for refinement. *R*-factors based on *F*^2^ are statistically about twice as large as those based on *F*, and *R*- factors based on ALL data will be even larger.

### Fractional atomic coordinates and isotropic or equivalent isotropic displacement parameters (Å^2^)

**Table:**

	*x*	*y*	*z*	*U*_iso_*/*U*_eq_	
C1	0.16111 (4)	0.4561 (2)	0.38426 (6)	0.0375 (3)	
H1	0.1730	0.5960	0.3771	0.045*	
C2	0.18453 (7)	0.1206 (3)	0.44221 (7)	0.0596 (5)	
H2A	0.2083	0.0676	0.4671	0.072*	
H2B	0.1601	0.0348	0.4455	0.072*	
C3	0.19346 (5)	0.1053 (3)	0.38717 (6)	0.0426 (3)	
C4	0.11402 (4)	0.4644 (2)	0.36653 (6)	0.0384 (3)	
C5	0.09284 (6)	0.6505 (3)	0.37624 (7)	0.0524 (4)	
H5	0.1078	0.7693	0.3913	0.063*	
C6	0.04944 (6)	0.6605 (4)	0.36362 (8)	0.0656 (5)	
H6	0.0355	0.7856	0.3706	0.079*	
C7	0.02702 (6)	0.4875 (4)	0.34099 (8)	0.0682 (6)	
H7	−0.0021	0.4951	0.3324	0.082*	
C8	0.04765 (6)	0.3011 (4)	0.33086 (8)	0.0651 (5)	
H8	0.0325	0.1834	0.3154	0.078*	
C9	0.09108 (5)	0.2897 (3)	0.34371 (7)	0.0514 (4)	
H9	0.1048	0.1639	0.3370	0.062*	
C10	0.18622 (4)	0.3123 (3)	0.30503 (5)	0.0373 (3)	
C11	0.17313 (5)	0.1509 (3)	0.26884 (7)	0.0511 (4)	
H11	0.1613	0.0246	0.2790	0.061*	
C12	0.17791 (7)	0.1806 (4)	0.21694 (8)	0.0693 (6)	
H12	0.1690	0.0736	0.1923	0.083*	
C13	0.19558 (7)	0.3659 (4)	0.20187 (8)	0.0704 (6)	
H13	0.1989	0.3836	0.1673	0.084*	
C14	0.20842 (6)	0.5253 (4)	0.23787 (7)	0.0629 (5)	
H14	0.2203	0.6510	0.2275	0.075*	
C15	0.20371 (5)	0.4996 (3)	0.28963 (6)	0.0471 (4)	
H15	0.2123	0.6083	0.3139	0.057*	
C16	0.08432 (5)	0.0210 (3)	0.54421 (6)	0.0402 (3)	
H16	0.0716	−0.1127	0.5541	0.048*	
C17	0.05773 (6)	0.3511 (3)	0.48483 (7)	0.0577 (5)	
H17A	0.0809	0.4348	0.4764	0.069*	
H17B	0.0321	0.3998	0.4628	0.069*	
C18	0.05479 (5)	0.3792 (3)	0.54178 (6)	0.0426 (3)	
C19	0.13159 (4)	−0.0004 (2)	0.55810 (5)	0.0371 (3)	
C20	0.14969 (5)	−0.1974 (3)	0.55010 (6)	0.0455 (4)	
H20	0.1327	−0.3128	0.5370	0.055*	
C21	0.19270 (5)	−0.2246 (3)	0.56134 (7)	0.0513 (4)	
H21	0.2044	−0.3573	0.5554	0.062*	
C22	0.21819 (5)	−0.0559 (3)	0.58124 (7)	0.0521 (4)	
H22	0.2471	−0.0746	0.5890	0.063*	
C23	0.20071 (5)	0.1409 (3)	0.58958 (7)	0.0536 (4)	
H23	0.2179	0.2552	0.6031	0.064*	
C24	0.15749 (5)	0.1688 (3)	0.57782 (7)	0.0469 (4)	
H24	0.1459	0.3024	0.5833	0.056*	
C25	0.07029 (4)	0.1912 (3)	0.62627 (6)	0.0406 (3)	
C26	0.08743 (5)	0.3596 (3)	0.65831 (7)	0.0531 (4)	
H26	0.0975	0.4818	0.6439	0.064*	
C27	0.08940 (6)	0.3447 (4)	0.71192 (8)	0.0694 (6)	
H27	0.1007	0.4575	0.7336	0.083*	
C28	0.07459 (7)	0.1627 (5)	0.73317 (8)	0.0745 (6)	
H28	0.0758	0.1535	0.7692	0.089*	
C29	0.05807 (7)	−0.0052 (4)	0.70153 (8)	0.0704 (6)	
H29	0.0485	−0.1283	0.7162	0.084*	
C30	0.05565 (5)	0.0088 (3)	0.64786 (7)	0.0537 (4)	
H30	0.0442	−0.1042	0.6264	0.064*	
N1	0.18173 (3)	0.28663 (19)	0.35879 (4)	0.0351 (3)	
N2	0.06799 (4)	0.2018 (2)	0.57083 (5)	0.0397 (3)	
O1	0.20931 (4)	−0.0527 (2)	0.37092 (5)	0.0597 (3)	
O2	0.04255 (4)	0.54461 (19)	0.55954 (5)	0.0579 (3)	
S1	0.175220 (14)	0.39920 (8)	0.454463 (16)	0.05330 (15)	
S2	0.065806 (16)	0.06983 (9)	0.474346 (18)	0.06317 (17)	

### Atomic displacement parameters (Å^2^)

**Table:**

	*U*^11^	*U*^22^	*U*^33^	*U*^12^	*U*^13^	*U*^23^
C1	0.0426 (7)	0.0352 (7)	0.0370 (7)	−0.0009 (6)	0.0132 (6)	−0.0069 (6)
C2	0.0781 (12)	0.0579 (11)	0.0449 (9)	0.0001 (9)	0.0168 (9)	0.0069 (8)
C3	0.0441 (8)	0.0392 (8)	0.0443 (8)	−0.0014 (6)	0.0074 (6)	0.0008 (6)
C4	0.0418 (7)	0.0411 (8)	0.0348 (7)	0.0036 (6)	0.0132 (6)	0.0014 (6)
C5	0.0591 (10)	0.0454 (9)	0.0545 (10)	0.0119 (8)	0.0146 (8)	−0.0003 (8)
C6	0.0604 (11)	0.0722 (13)	0.0668 (12)	0.0306 (10)	0.0180 (9)	0.0060 (10)
C7	0.0427 (9)	0.1013 (17)	0.0607 (11)	0.0169 (10)	0.0097 (8)	0.0031 (11)
C8	0.0455 (9)	0.0795 (14)	0.0704 (13)	−0.0051 (9)	0.0104 (8)	−0.0143 (11)
C9	0.0426 (8)	0.0533 (10)	0.0592 (10)	0.0017 (7)	0.0114 (7)	−0.0104 (8)
C10	0.0342 (6)	0.0433 (8)	0.0352 (7)	0.0057 (6)	0.0079 (5)	−0.0031 (6)
C11	0.0538 (9)	0.0532 (10)	0.0463 (9)	−0.0014 (7)	0.0085 (7)	−0.0137 (8)
C12	0.0784 (13)	0.0829 (15)	0.0450 (10)	0.0099 (11)	0.0065 (9)	−0.0251 (10)
C13	0.0863 (14)	0.0896 (16)	0.0386 (9)	0.0114 (12)	0.0202 (9)	0.0010 (10)
C14	0.0737 (12)	0.0718 (13)	0.0473 (10)	−0.0001 (10)	0.0222 (9)	0.0105 (9)
C15	0.0515 (9)	0.0504 (9)	0.0408 (8)	−0.0016 (7)	0.0122 (7)	−0.0012 (7)
C16	0.0413 (7)	0.0379 (8)	0.0423 (8)	−0.0002 (6)	0.0096 (6)	−0.0062 (6)
C17	0.0537 (9)	0.0693 (12)	0.0489 (10)	0.0055 (9)	0.0055 (8)	0.0070 (9)
C18	0.0365 (7)	0.0410 (8)	0.0500 (9)	−0.0020 (6)	0.0067 (6)	−0.0005 (7)
C19	0.0411 (7)	0.0376 (8)	0.0336 (7)	0.0021 (6)	0.0093 (5)	0.0006 (6)
C20	0.0508 (8)	0.0392 (8)	0.0456 (8)	0.0029 (6)	0.0065 (7)	−0.0051 (7)
C21	0.0535 (9)	0.0496 (10)	0.0504 (9)	0.0174 (7)	0.0079 (7)	0.0010 (8)
C22	0.0414 (8)	0.0668 (11)	0.0474 (9)	0.0074 (8)	0.0057 (7)	0.0061 (8)
C23	0.0443 (8)	0.0546 (10)	0.0611 (11)	−0.0075 (7)	0.0073 (7)	−0.0021 (8)
C24	0.0471 (8)	0.0391 (8)	0.0553 (10)	0.0004 (6)	0.0116 (7)	−0.0029 (7)
C25	0.0351 (6)	0.0466 (9)	0.0405 (8)	0.0064 (6)	0.0079 (6)	−0.0038 (6)
C26	0.0509 (9)	0.0543 (10)	0.0531 (10)	0.0032 (7)	0.0069 (7)	−0.0110 (8)
C27	0.0635 (11)	0.0880 (15)	0.0518 (11)	0.0155 (11)	−0.0033 (9)	−0.0251 (11)
C28	0.0700 (12)	0.1132 (19)	0.0399 (10)	0.0204 (13)	0.0089 (9)	0.0033 (11)
C29	0.0716 (13)	0.0898 (16)	0.0521 (11)	0.0036 (11)	0.0171 (9)	0.0159 (11)
C30	0.0528 (9)	0.0592 (11)	0.0500 (10)	−0.0004 (8)	0.0114 (7)	0.0029 (8)
N1	0.0375 (6)	0.0343 (6)	0.0348 (6)	0.0018 (5)	0.0099 (5)	−0.0036 (5)
N2	0.0405 (6)	0.0379 (7)	0.0413 (7)	0.0023 (5)	0.0094 (5)	−0.0027 (5)
O1	0.0756 (8)	0.0412 (7)	0.0634 (8)	0.0151 (6)	0.0151 (6)	0.0017 (6)
O2	0.0582 (7)	0.0416 (7)	0.0725 (9)	0.0086 (5)	0.0076 (6)	0.0006 (6)
S1	0.0549 (2)	0.0697 (3)	0.0357 (2)	0.0024 (2)	0.00903 (17)	−0.01146 (19)
S2	0.0619 (3)	0.0795 (4)	0.0439 (3)	0.0126 (2)	−0.0019 (2)	−0.0167 (2)

### Geometric parameters (Å, º)

**Table:**

C1—N1	1.4641 (18)	C16—N2	1.4638 (19)
C1—C4	1.515 (2)	C16—C19	1.516 (2)
C1—S1	1.8328 (17)	C16—S2	1.8315 (17)
C1—H1	0.9800	C16—H16	0.9800
C2—C3	1.511 (2)	C17—C18	1.509 (2)
C2—S1	1.791 (2)	C17—S2	1.790 (2)
C2—H2A	0.9700	C17—H17A	0.9700
C2—H2B	0.9700	C17—H17B	0.9700
C3—O1	1.216 (2)	C18—O2	1.219 (2)
C3—N1	1.360 (2)	C18—N2	1.359 (2)
C4—C5	1.387 (2)	C19—C24	1.383 (2)
C4—C9	1.386 (2)	C19—C20	1.386 (2)
C5—C6	1.388 (3)	C20—C21	1.383 (2)
C5—H5	0.9300	C20—H20	0.9300
C6—C7	1.368 (3)	C21—C22	1.375 (3)
C6—H6	0.9300	C21—H21	0.9300
C7—C8	1.383 (3)	C22—C23	1.378 (3)
C7—H7	0.9300	C22—H22	0.9300
C8—C9	1.389 (2)	C23—C24	1.391 (2)
C8—H8	0.9300	C23—H23	0.9300
C9—H9	0.9300	C24—H24	0.9300
C10—C11	1.386 (2)	C25—C30	1.382 (2)
C10—C15	1.382 (2)	C25—C26	1.387 (2)
C10—N1	1.4378 (18)	C25—N2	1.430 (2)
C11—C12	1.396 (3)	C26—C27	1.385 (3)
C11—H11	0.9300	C26—H26	0.9300
C12—C13	1.371 (3)	C27—C28	1.379 (4)
C12—H12	0.9300	C27—H27	0.9300
C13—C14	1.373 (3)	C28—C29	1.374 (4)
C13—H13	0.9300	C28—H28	0.9300
C14—C15	1.389 (2)	C29—C30	1.384 (3)
C14—H14	0.9300	C29—H29	0.9300
C15—H15	0.9300	C30—H30	0.9300
			
N1—C1—C4	113.81 (12)	C19—C16—H16	108.6
N1—C1—S1	104.96 (10)	S2—C16—H16	108.6
C4—C1—S1	111.61 (10)	C18—C17—S2	107.33 (13)
N1—C1—H1	108.8	C18—C17—H17A	110.2
C4—C1—H1	108.8	S2—C17—H17A	110.2
S1—C1—H1	108.8	C18—C17—H17B	110.2
C3—C2—S1	107.19 (12)	S2—C17—H17B	110.2
C3—C2—H2A	110.3	H17A—C17—H17B	108.5
S1—C2—H2A	110.3	O2—C18—N2	124.19 (16)
C3—C2—H2B	110.3	O2—C18—C17	123.34 (16)
S1—C2—H2B	110.3	N2—C18—C17	112.46 (14)
H2A—C2—H2B	108.5	C24—C19—C20	118.54 (14)
O1—C3—N1	124.89 (15)	C24—C19—C16	122.89 (14)
O1—C3—C2	122.89 (15)	C20—C19—C16	118.57 (14)
N1—C3—C2	112.22 (14)	C21—C20—C19	120.90 (15)
C5—C4—C9	118.82 (15)	C21—C20—H20	119.5
C5—C4—C1	118.53 (14)	C19—C20—H20	119.5
C9—C4—C1	122.56 (13)	C20—C21—C22	120.17 (15)
C4—C5—C6	120.52 (18)	C20—C21—H21	119.9
C4—C5—H5	119.7	C22—C21—H21	119.9
C6—C5—H5	119.7	C23—C22—C21	119.70 (15)
C7—C6—C5	120.33 (18)	C23—C22—H22	120.2
C7—C6—H6	119.8	C21—C22—H22	120.2
C5—C6—H6	119.8	C22—C23—C24	120.14 (16)
C6—C7—C8	119.88 (17)	C22—C23—H23	119.9
C6—C7—H7	120.1	C24—C23—H23	119.9
C8—C7—H7	120.1	C19—C24—C23	120.55 (15)
C7—C8—C9	120.04 (19)	C19—C24—H24	119.7
C7—C8—H8	120.0	C23—C24—H24	119.7
C9—C8—H8	120.0	C30—C25—C26	120.09 (16)
C4—C9—C8	120.41 (17)	C30—C25—N2	119.07 (14)
C4—C9—H9	119.8	C26—C25—N2	120.84 (15)
C8—C9—H9	119.8	C27—C26—C25	119.63 (19)
C11—C10—C15	120.12 (15)	C27—C26—H26	120.2
C11—C10—N1	120.44 (15)	C25—C26—H26	120.2
C15—C10—N1	119.43 (13)	C28—C27—C26	120.0 (2)
C10—C11—C12	119.10 (18)	C28—C27—H27	120.0
C10—C11—H11	120.5	C26—C27—H27	120.0
C12—C11—H11	120.5	C29—C28—C27	120.43 (19)
C13—C12—C11	120.64 (18)	C29—C28—H28	119.8
C13—C12—H12	119.7	C27—C28—H28	119.8
C11—C12—H12	119.7	C28—C29—C30	120.0 (2)
C12—C13—C14	119.99 (18)	C28—C29—H29	120.0
C12—C13—H13	120.0	C30—C29—H29	120.0
C14—C13—H13	120.0	C25—C30—C29	119.84 (19)
C13—C14—C15	120.30 (19)	C25—C30—H30	120.1
C13—C14—H14	119.9	C29—C30—H30	120.1
C15—C14—H14	119.9	C3—N1—C10	123.39 (12)
C10—C15—C14	119.85 (16)	C3—N1—C1	116.99 (12)
C10—C15—H15	120.1	C10—N1—C1	119.50 (12)
C14—C15—H15	120.1	C18—N2—C25	123.43 (13)
N2—C16—C19	112.93 (12)	C18—N2—C16	117.51 (13)
N2—C16—S2	105.00 (10)	C25—N2—C16	118.80 (12)
C19—C16—S2	112.89 (10)	C2—S1—C1	91.65 (8)
N2—C16—H16	108.6	C17—S2—C16	92.37 (8)
			
S1—C2—C3—O1	164.39 (14)	N2—C25—C26—C27	−179.78 (15)
S1—C2—C3—N1	−15.65 (18)	C25—C26—C27—C28	0.4 (3)
N1—C1—C4—C5	−162.23 (14)	C26—C27—C28—C29	0.4 (3)
S1—C1—C4—C5	79.20 (16)	C27—C28—C29—C30	−0.9 (3)
N1—C1—C4—C9	21.5 (2)	C26—C25—C30—C29	0.1 (3)
S1—C1—C4—C9	−97.08 (15)	N2—C25—C30—C29	179.28 (16)
C9—C4—C5—C6	0.4 (3)	C28—C29—C30—C25	0.7 (3)
C1—C4—C5—C6	−176.01 (16)	O1—C3—N1—C10	0.9 (2)
C4—C5—C6—C7	−0.6 (3)	C2—C3—N1—C10	−179.02 (14)
C5—C6—C7—C8	0.3 (3)	O1—C3—N1—C1	176.89 (15)
C6—C7—C8—C9	0.1 (3)	C2—C3—N1—C1	−3.07 (19)
C5—C4—C9—C8	0.0 (3)	C11—C10—N1—C3	47.6 (2)
C1—C4—C9—C8	176.27 (17)	C15—C10—N1—C3	−132.45 (15)
C7—C8—C9—C4	−0.3 (3)	C11—C10—N1—C1	−128.26 (15)
C15—C10—C11—C12	0.1 (2)	C15—C10—N1—C1	51.69 (18)
N1—C10—C11—C12	−179.99 (15)	C4—C1—N1—C3	−102.68 (15)
C10—C11—C12—C13	0.5 (3)	S1—C1—N1—C3	19.64 (15)
C11—C12—C13—C14	−0.6 (3)	C4—C1—N1—C10	73.44 (16)
C12—C13—C14—C15	0.2 (3)	S1—C1—N1—C10	−164.24 (10)
C11—C10—C15—C14	−0.5 (2)	O2—C18—N2—C25	−2.1 (2)
N1—C10—C15—C14	179.58 (15)	C17—C18—N2—C25	176.81 (13)
C13—C14—C15—C10	0.4 (3)	O2—C18—N2—C16	−176.11 (14)
S2—C17—C18—O2	−167.49 (13)	C17—C18—N2—C16	2.75 (19)
S2—C17—C18—N2	13.64 (17)	C30—C25—N2—C18	135.69 (16)
N2—C16—C19—C24	−21.5 (2)	C26—C25—N2—C18	−45.1 (2)
S2—C16—C19—C24	97.45 (16)	C30—C25—N2—C16	−50.32 (19)
N2—C16—C19—C20	159.33 (14)	C26—C25—N2—C16	128.84 (15)
S2—C16—C19—C20	−81.74 (16)	C19—C16—N2—C18	106.20 (15)
C24—C19—C20—C21	−0.2 (2)	S2—C16—N2—C18	−17.21 (15)
C16—C19—C20—C21	178.98 (15)	C19—C16—N2—C25	−68.14 (17)
C19—C20—C21—C22	0.7 (3)	S2—C16—N2—C25	168.44 (10)
C20—C21—C22—C23	−0.5 (3)	C3—C2—S1—C1	22.65 (14)
C21—C22—C23—C24	−0.1 (3)	N1—C1—S1—C2	−23.76 (11)
C20—C19—C24—C23	−0.4 (2)	C4—C1—S1—C2	99.98 (12)
C16—C19—C24—C23	−179.55 (15)	C18—C17—S2—C16	−19.83 (12)
C22—C23—C24—C19	0.5 (3)	N2—C16—S2—C17	20.77 (11)
C30—C25—C26—C27	−0.6 (2)	C19—C16—S2—C17	−102.66 (12)

### Hydrogen-bond geometry (Å, º)

**Table:**

*D*—H···*A*	*D*—H	H···*A*	*D*···*A*	*D*—H···*A*
C15—H15···O1^i^	0.93	2.58	3.470 (2)	160
C1—H1···O1^i^	0.98	2.49	3.466 (2)	172
C16—H16···O2^ii^	0.98	2.34	3.301 (3)	168
C17—H17*B*···O2^iii^	0.97	2.41	3.313 (3)	155

Symmetry codes: (i) *x*, *y*+1, *z*; (ii) *x*, *y*−1, *z*; (iii) −*x*, −*y*+1, −*z*+1.
